# Characterisation of pellicle-forming ability in clinical carbapenem-resistant *Acinetobacter baumannii*

**DOI:** 10.7717/peerj.15304

**Published:** 2023-05-15

**Authors:** Heng Kang Ng, Suat Moi Puah, Cindy Shuan Ju Teh, Nuryana Idris, Kek Heng Chua

**Affiliations:** 1 Department of Biomedical Science, Faculty of Medicine, Universiti Malaya, Kuala Lumpur, Malaysia; 2 Department of Medical Microbiology, Faculty of Medicine, Universiti Malaya, Kuala Lumpur, Malaysia

**Keywords:** Biomass, Carbapenem-resistant Acinetobacter baumannii, GacA, GacS, Pellicle

## Abstract

**Background:**

*Acinetobacter baumannii* was reported to have resistance towards carbapenems and the ability to form an air-liquid biofilm (pellicle) which contributes to their virulence. The GacSA two-component system has been previously shown to play a role in pellicle formation. Therefore, this study aims to detect the presence of *gacA* and *gacS* genes in carbapenem-resistant *Acinetobacter baumannii* (CRAB) isolates recovered from patients in intensive care units and to investigate their pellicle forming ability.

**Methods:**

The *gacS* and *gacA* genes were screened in 96 clinical CRAB isolates using PCR assay. Pellicle formation assay was performed in Mueller Hinton medium and Luria Bertani medium using borosilicate glass tubes and polypropylene plastic tubes. The biomass of the pellicle was quantitated using the crystal violet staining assay. The selected isolates were further assessed for their motility using semi-solid agar and monitored in real-time using real-time cell analyser (RTCA).

**Results:**

All 96 clinical CRAB isolates carried the *gacS* and *gacA* genes, however, only four isolates (AB21, AB34, AB69 and AB97) displayed the ability of pellicle-formation phenotypically. These four pellicle-forming isolates produced robust pellicles in Mueller Hinton medium with better performance in borosilicate glass tubes in which biomass with OD_570_ ranging from 1.984 ± 0.383 to 2.272 ± 0.376 was recorded. The decrease in cell index starting from 13 hours obtained from the impedance-based RTCA showed that pellicle-forming isolates had entered the growth stage of pellicle development.

**Conclusion:**

These four pellicle-forming clinical CRAB isolates could be potentially more virulent, therefore further investigation is warranted to provide insights into their pathogenic mechanisms.

## Introduction

*Acinetobacter baumannii* is an opportunistic Gram-negative, obligate aerobic human pathogen that predominantly targets immunocompromised patients, causing a therapeutic impasse, especially during this heavy hospitalisation of coronavirus disease-19 (COVID-19) era (Harding, Hennon & Feldman, 2018; Rangel, Chagas & De-Simone, 2021). It displayed unforeseen rates of multidrug, extensive drug and pan-drug resistance due to its outstanding ability to acquire or upregulate diverse resistance determinants (Harding, Hennon & Feldman, 2018; Vrancianu et al., 2020). The multidrug-resistance nosocomial *A. baumannii* is reported to have an overall prevalence ranging from 40% to 95% among patients with hospital-acquired infections such as ventilator-associated pneumonia and bloodstream infections (Mohd Sazlly Lim et al., 2019). The World Health Organisation even declared carbapenem-resistant *A. baumannii* (CRAB) as critical priority bacteria for new antibiotic research, discovery and development (Sarshar et al., 2021).

Moreover, *A. baumannii* can survive in harsh clinical settings such as the presence of disinfectants and prolonged periods of desiccation (Krasauskas et al., 2019). This phenomenon could be due to their ability to attach and form biofilms on abiotic and biotic surfaces. Biofilms are the assemblage of surface-associated microbial cells that is enclosed in a multilayer extracellular polymeric substance matrix such as exopolysaccharides, proteins, nucleic acids and other substances. They are not continuous monolayer surface deposits but rather very heterogeneous, containing microcolonies of bacterial cells encased in the extracellular polymeric substance matrix and separated from other microcolonies by interstitial voids such as water channels (Donlan, 2002; Kentache et al., 2017). This extracellular matrix provides a protective layer against the penetration of antibiotics and creates an optimal environment for genetic material exchange between the microorganisms (Kentache et al., 2017).

Biofilms can be developed at the solid-liquid, liquid-liquid and air-liquid interfaces (Mandakhalikar, 2019). The formation of solid-liquid biofilm in bacteria is well studied yet the air-liquid biofilm formation is less investigated as it is a selectively advantageous niche for bacteria to gain direct access to oxygen from the air and nutrients from the liquid media simultaneously, such as *Pseudomonas aeruginosa and Shewanella oneidensis* (Pang, Yang & Yuk, 2017; Wu & Jin, 2019)*. Acinetobacter baumannii*, in particular, was also reported to have the capability to develop an air-liquid interface floating biofilm, known as a pellicle (Oh & Han, 2020). Studies reported that pellicle-forming *Acinetobacter* species, such as *A. baumannii* and *A. nosocomialis* are mainly associated with nosocomial infections, therefore, suggesting a correlation between the pellicle formation feature and their virulence potential (Kentache et al., 2017; Marti et al., 2011; Peleg et al., 2008).

The regulation of biofilm formation was reported to be influenced by sensing bacterial cell density and the presence of different nutrients and cations through the regulatory two-component systems (TCSs) (Lin, Lin & Lan, 2020). *A. baumannii* possesses several TCSs including AdeRS, BaeSR, PmrAB, GacSA, BfmRS, and A1S_2811 that were responsible to influence their pathogenic potentials (De Silva & Kumar, 2019). Among these TCSs, the GacSA TCS was reported to play a significant role for cell growth and adaptation to complex environmental changes *via* regulates gene expression at the transcription and post-transcriptional levels (Song, Li & Wang, 2023). Moreover, the GacSA TCS reported to be responsible in participating in biofilm formation in *Pseudomonas aeruginosa.* In *P. aeruginosa* PA14, the deletion of *gacS* was observed to result in decreased of biofilm development and higher susceptibility to antibiotics (Davies et al., 2007). Therefore, the GacSA-mediated biofilm formation that induced antibiotic resistance has important research value hence warrant for more studies, including in *Acinetobacter baumannii.* The GacSA TCS, encoded by the *gacA* and *gacS* genes has a high degree of the conservation at nucleotide level (99–100%) in the nature of *A. baumannii* (De Silva & Kumar, 2019). Particularly, the GacA was reported to achieve a four-fold increase in protein yield in four-day pellicle cells using a proteome study, suggesting that the GacSA TCS has an association with pellicle formation (Kentache et al., 2017). Besides, the *gacA* gene has also been reported to be associated with multiple aspects of *A. baumannii* infections together with the *gacS* gene using a murine model of pneumonia *via* a combination of transposon mutagenesis and massively parallel next-generation sequencing approach (Wang et al., 2014). Another group of researchers also demonstrated that the avirulent phenotype of deleted *gacS* gene caused a greatly decreased bacteria load in the kidney, liver and spleen of a mouse septicaemia model at 12 h post-infection (Cerqueira et al., 2014).

Thus far, the knowledge on the involvement of GacSA TCS for pellicle formation in *A. baumannii* is based on a study using a type strain of *A. baumannii* ATCC 17978 (Kentache et al., 2017)*.* Therefore, it is of interest to study *gacS* and *gacA* genes in pellicle formation using clinical CRAB isolates. Overall, the objectives of this study are (i) to detect the presence of *gacA* and *gacS* gene using polymerase chain reaction assay in clinical CRAB isolates, (ii) to investigate their ability to form pellicles using pellicle formation assay (iii) to study the selected pellicle-forming strains using surface-associated motility assay and impedance-based cell analysis with the xCELLigence Real-Time Cell Analyzer (RTCA).

## Materials & Methods

### Bacterial strains and growth conditions

A total of 96 clinical CRAB isolates used in this study were collected from intensive care units, at the University Malaya Medical Center from the year 2015 to 2016 (Woon et al., 2021). The bacteria isolates were cryopreserved in Luria Bertani (LB) broth (BD Difco, Franklin Lakes, NJ, USA) with 20% (v/v) glycerol at −80 °C. All isolates were streaked on LB agar to obtain a pure colony for further investigation.

### Genomic DNA extraction

The genomic DNA of isolates was extracted from overnight cultures using the in-house boiling method (Puah et al., 2018). In brief, the pellet of 1.5 mL overnight bacteria culture was obtained through centrifugation at 13,000 rpm for 1 min and washed twice with distilled water. The supernatant was then discarded and 100 µL of sterile distilled water was added to resuspend the pellet and followed by a boiling period of 10 min at 95 °C. The lysed bacterial cell was then incubated on ice for 10 min and centrifuged at 13,000 rpm for 1 min. The supernatants were collected in a new microcentrifuge tube as extracted DNA samples. The quantity and quality of extracted total genomic DNA samples were measured spectrophotometrically and stored at −20 °C for further investigation.

### Genotypic screening of *gacA* and *gacS* gene using polymerase chain reaction assay

All isolates were subject to a direct polymerase chain reaction amplification assay to detect the presence of the *gacA* and *gacS* genes. The housekeeping gene, *rpoD* was used as a positive control for *A. baumannii*. The primer pairs were designed based on the reference *Acinetobacter baumannii* strains AB5075-UW (CP008706.1) as follows: (i) *gacA*_F (5′-TGCTATCGTTCGCCAACAACATCC-3′) & *gacA*_R (5′-CATTGCAACTTGCATTTCCCGTTCC-3′) (ii) *gacS*_F (5′- ATTCTCGCCGCTGGATTGCACC-3′) & *gacS*_R (5′- TGCATCGCCTTGCGAAAACGAC-3′) and (iii) *rpoD*_F (5′- GGCAGCAGCTTCTTCTTCAGCAAC-3′) & *rpoD*_R (5′- GCCGGACTCGATTACTGAAAGCG-3′). The expected amplicon size are: 355 bp (CP008706.1: 3,698,889–3,699,243) for *gacA* gene; 830 bp (CP008706.1: 3,339,672–3,340,501) for *gacS* gene and 160 bp (CP008706.1: 842,931–843,090) for *rpoD* gene.

### Phenotypic assessment of pellicle formation using pellicle formation assay

Bacteria cultures grown to log phase, OD_600_ of 0.6 and diluted into OD_600_ of 0.1 of different broths were aliquoted 2.5 mL into different materials as followed: (i) borosilicate glass tube with Mueller Hinton (MH) broth (BD Difco, Franklin Lakes, NJ, USA), (ii) borosilicate glass tube with LB broth, (iii) polypropylene plastic tube with MH broth and (iv) polypropylene plastic tube with LB broth. The cultures were then incubated in static at 37 °C for 48 h in the dark. The visual examination of pellicle formation was conducted at 48 h.

### Quantification of pellicle biomass using crystal violet staining assay

Crystal violet assay with modification from Biswas & Mettlach (2019) and O’Toole & Kolter (1998) protocols was used in this study. Briefly, the pellicle will form on top of the culture, far from the bottom of the tubes. Therefore, the supernatant of cultures was discarded carefully without removing the pellicle. Next, the bottom of the tubes was vigorously rinsed with sterile phosphate-buffered saline (PBS) solution to remove any sediments and followed by staining using 3 mL of 0.2% (w/v) crystal violet solution for 15 min at room temperature. Then, the crystal violet solution was discarded and washed with 3 mL of PBS solution to remove any crystal violet solution residue. The PBS solution was then discarded and the stained pellicle was left to air dry for 30 min. A total of 3 mL of 30% (v/v) acetic acid was added and incubated for 30 min for solubilisation. The biomass was then extracted and measured for its optical density reading at 570 nm. Three independent experiments were performed in triplicates and the average OD_570_ values were calculated. The results between pellicle-forming isolates and non-pellicle-forming isolates were analysed with Dunnett’s multiple comparison test of one-way analysis of variance (ANOVA) test by using GraphPad Prism version 8.0.2 (GraphPad Software, USA). The differences at the *p*-value < 0.05 were considered statistically significant.

### Surface-associated motility assay

The pellicle-forming and non-pellicle-forming isolates were selected and subjected to surface-associated motility assay following lab-established protocol (Lau et al., 2021). Freshly prepared 25 mL nutrient broth containing 0.5% (w/v) Bacto™ agar (BD Difco, Franklin Lakes, NJ, USA) in 90 mm petri dishes and air-dried in laminar flow for 30 min were used as motility assay medium. Briefly, bacterial cultures were grown to log phase at OD_600_ of 0.6 and subjected to dilution of OD_600_ of 0.1. Two microliters of diluted bacterial cultures were inoculated in the centre of the motility agar. The plates were incubated at 37 °C for 24 h. Bacterial migration distance from the original inoculation site was measured. Motile *Salmonella typhimurium* ATCC 14028 and non-motile *Klebsiella pneumoniae* ATCC 700603 were used as positive and negative controls, respectively (Kim & Surette, 2005; Lau et al., 2021). Three independent experiments were performed in triplicates and bacterial migration distance from the original inoculation site was measured. Surface-associated motility-positive isolates were defined as those isolates that showed a larger zone of the area around the site of inoculation than negative control *Klebsiella pneumoniae* ATCC 700603 (Kim & Surette, 2005). The results were analysed using Dunnett’s multiple comparisons ANOVA test using GraphPad Prism version 8.0.2 (GraphPad Software, San Diego, CA, USA). The differences at the *p*-value < 0.001 were considered statistically significant.

### Real-time monitoring of pellicle formation using impedance-based xCELLigence real-time cell analyser

The pellicle-forming isolates and non-pellicle-forming isolates were selected for further investigation in real-time using the impedance-based xCELLigence RTCA (Jones et al., 2023; Lau et al., 2023). Briefly, the bacterial cultures were grown to log phase at OD_600_ of 0.6 and subjected to dilution to OD_600_ of 0.1. A total of 100 µL diluted bacterial cultures were added to wells of E-Plate and 100 µL of MH medium was used as the background measurement. After that, the E-Plate was placed into the RCTA and incubated at 37 °C. The RTCA was programmed to record impedance measurements every 10 min over 48 h. Three independent experiments were performed in triplicates and the average cell index (CI) ± standard deviation was calculated.

## Results

### Genotypic screening of *gacA* and *gacS* gene using polymerase chain reaction assay

**Figure 1:**
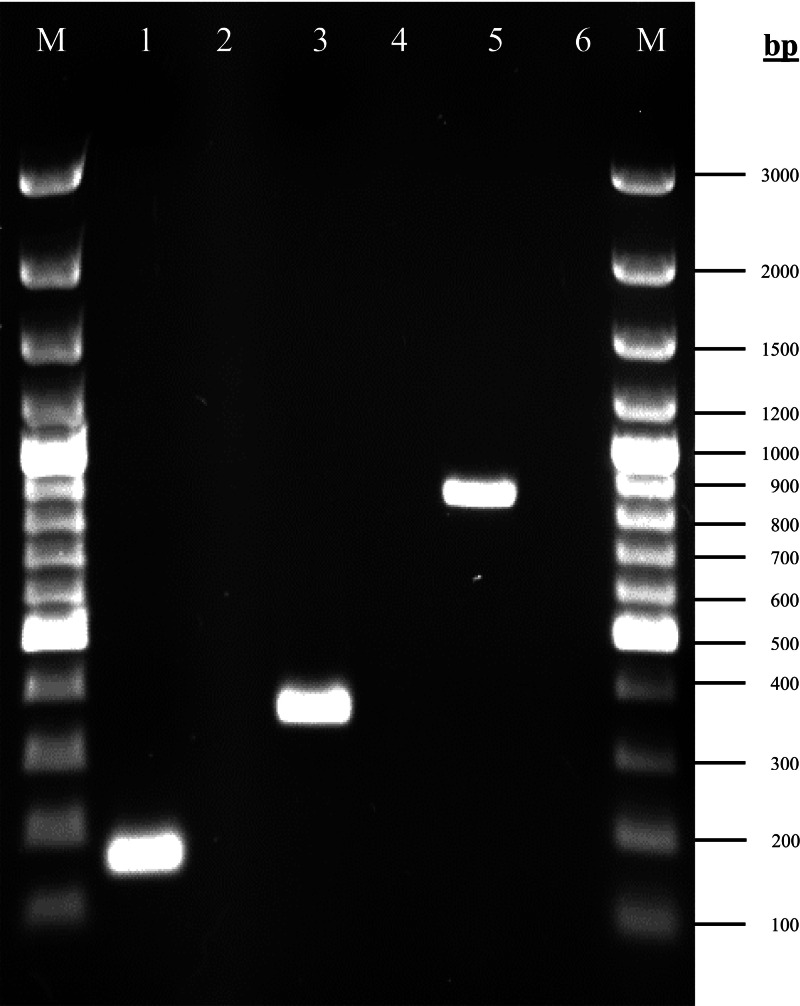
Agarose gel electrophoresis (1%) of PCR products for the *rpoD*, *gacA* and *gacS* gene fragments. Lane M: 100bp plus GeneRuler DNA ladder (Thermo Fisher Scientific, Waltham, MA, USA); lane 1: *rpoD* (160 bp); lane 3: *gacA* (355 bp); lane 5: *gacS* (830 bp); lanes 2, 4 & 6: non-template controls.

The amplified products of *rpoD*, *gacA* and *gacS* genes corresponding to the expected size are shown in Fig. 1. The *rpoD* gene was present in all isolates, confirming that all strains belong to *A. baumannii*. Both *gacA* and *gacS* genes were detected in 100% (96/96) isolates.

### Phenotypic assessment of pellicle formation in carbapenem-resistant *Acinetobacter baumannii*

**Figure 2:**
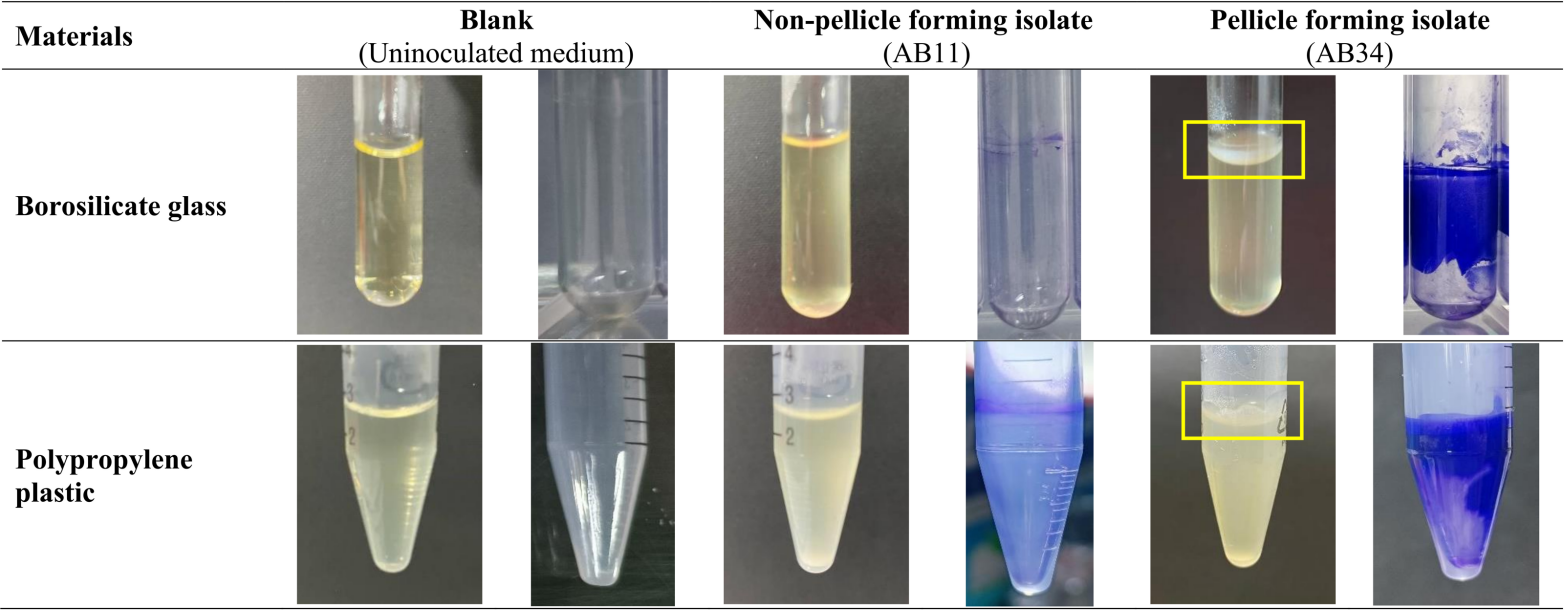
Visualisation of the pellicle formation in clinical carbapenem-resistant *Acinetobacter baumannii* isolates in Mueller-Hinton medium at 48 hours.

Of the 96 isolates, 4.2% (4/96) showed their ability to form pellicles at 48 h cultured in MH medium and LB medium using borosilicate glass tubes and polypropylene plastic tubes *via* visualisation. These four pellicle-forming isolates—AB21, AB34, AB69 and AB97 produce more robust pellicles in MH medium compared to LB medium in borosilicate glass tubes and polypropylene plastic tubes as shown in Fig. 2. This was further supported by quantification results of pellicle biomass whereby OD_570_ reading across four pellicle-forming isolates obtained from MH medium are higher than in LB medium (Table 1).

**Table 1:** The average crystal violet optical density reading of pellicle biomass of clinical carbapenem-resistant *Acinetobacter baumannii* isolates.

**Material**	**Medium**	**Crystal violet reading (OD**_**570**_ **)**				
		**Non-pellicle forming isolates**	**AB21**	**AB34**	**AB69**	**AB97**
**Borosilicate tube**	**Mueller Hinton**	1.047 ± 0.221	1.766 ± 0.132^****^	2.353 ± 0.202^****^	1.957 ± 0.384^****^	2.891 ± 0.481^****^
	**Luria Bertani**	1.092 ± 0.119	1.550 ± 0.173^*^	1.756 ± 0.074^***^	1.508 ± 0.085^*^	2.555 ± 0.264^****^
**Polypropylene tube**	**Mueller Hinton**	1.584 ± 0.185	1.699 ± 0.210^ns^	3.325 ± 0.945^****^	3.015 ± 0.773^****^	2.902 ± 0.195^****^
	**Luria Bertani**	1.152 ± 0.180	1.244 ± 0.333^ns^	1.164 ± 0.090^ns^	1.214 ± 0.175^ns^	2.597 ± 0.152^****^

**Notes.**

Footnote ns no significant differences. Statistically significant

* *p* < 0.05.

** *p* < 0.01.

*** *p* < 0.001.

**** *p* < 0.0001.

To examine the differences in the amount of pellicle biomass between these four pellicle-forming isolates (AB21, AB34, AB69 and AB97), three non-pellicle-forming isolates (AB11, AB20 and AB31) were randomly selected from respiratory-associated samples for comparative analysis. Overall, pellicle-forming isolates produced more robust biomass (OD_570_ ranging from 1.122 ± 0.159 to 3.203 ± 0.569) compared to the biomass from non-pellicle-forming isolates (an average OD_570_ ranging from 0.992 ± 0.111 to 1.697 ± 0.201) (Table 1). When comparing the impact of materials on pellicle growth among the four pellicle-forming isolates, all isolates significantly formed pellicles in borosilicate glass in both MH and LB mediums but were more robust in MH medium (*p* < 0.0001). However, the capability of pellicle formation in polypropylene tubes varies—three isolates (AB34, AB69 and AB97) formed pellicles in the MH medium (75%, 3/4) and only one isolate AB97 formed a pellicle in the LB medium (25%, 1/4).

### Surface-associated motility assay

**Figure 3:**
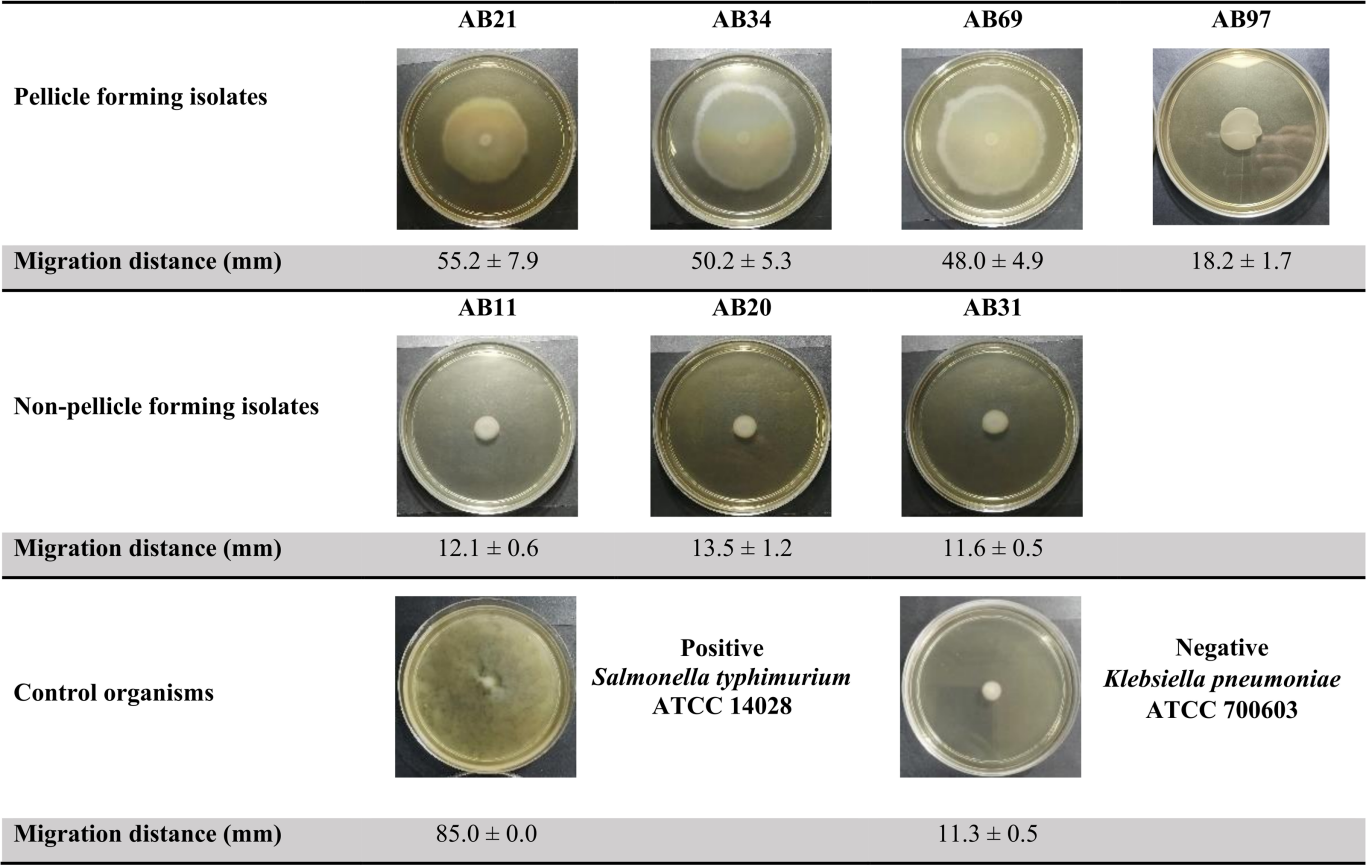
Surface-associated motility assay of selected clinical carbapenem-resistant *Acinetobacter baumannii* isolates on 0.5% Bacto™ agar.

The semi-solid agar worked well as both control organisms demonstrated the expected results (Fig. 3). The positive control motile *S. typhimurium* ATCC 14028 fully covered the whole semi-solid agar plate with a migration distance of 85 mm while the negative control non-motile *K. pneumoniae* ATCC 700603 showed a small distance of 11.3 mm, which was approximately 7.6 times less than the positive control. Three pellicle-forming clinical CRAB isolates (AB21, AB34 and AB69) exhibited significant surface-associated motility with distances ranging from 48.0 to 55.2 mm (*p* < 0.001) and AB97 exhibited fairly surface-associated motility with a distance of 18.2 mm (*p*-value = 0.0021) compared to the average migrating distance (12.4 mm) of non-pellicle-forming isolates (AB11, AB20 and AB31) (Fig. 3).

### Real-time monitoring of pellicle formation using impedance-based xCELLigence real-time cell analyser

**Figure 4:**
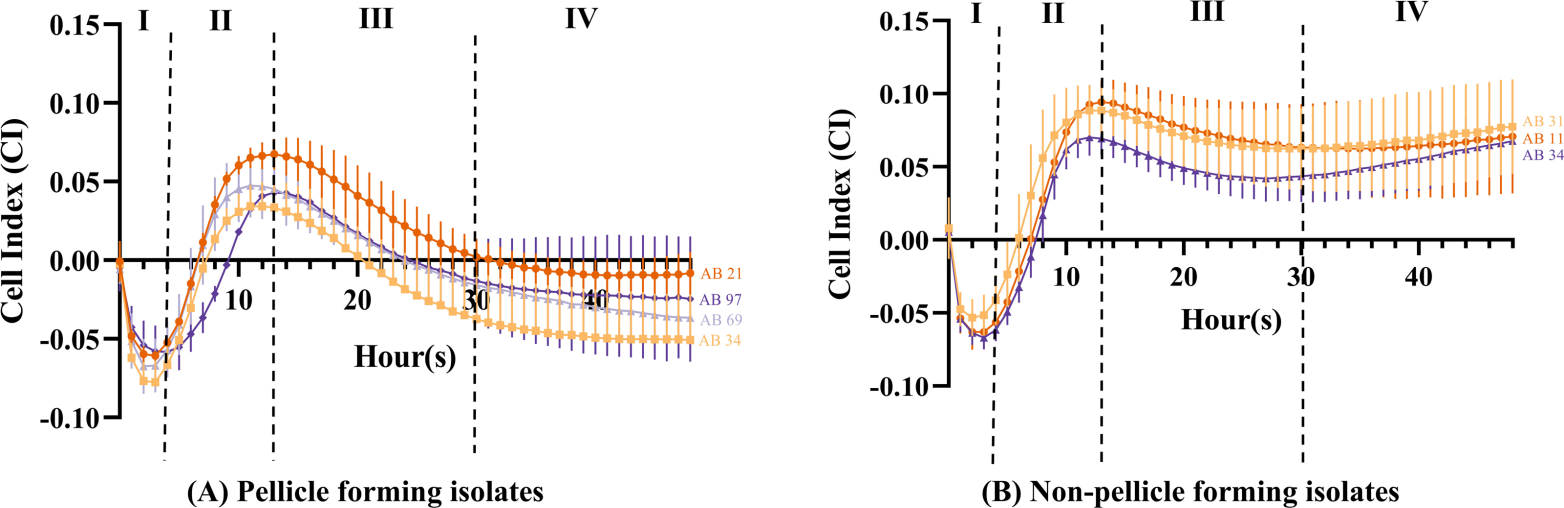
Cell index measured using impedance-based xCELLigence real-time cell analyser for monitoring pellicle formation of *Acinetobacter baumannii* in real-time for 48 hours. Cell index values represent average ± standard deviation of three independent experiments.

The cell index value obtained can be distinguished into four different stages. From 0 to 4 h (Stage I), the CI values of all isolates (Figs. 4A and 4B) showed a steep decrease with an approximate CI value of −0.05, but the CI values began to increase steadily during 4 to 13 h (Stage II). At 11 h, pellicle-forming isolates AB34 and AB69 reached their maximum CI values of 0.034 and 0.047 respectively, while another two pellicle-forming isolates AB21 and AB97 reached maximum CI values of 0.067 and 0.043 at 13 h (Fig. 4A). For non-pellicle-forming strains, AB20 and AB31 reached their maximum CI values of 0.070 and 0.088 at 12 h while AB11 was observed at 13 h with a CI value of 0.094 (Fig. 4B). From 13 h onward to 30 h (Stage III), the CI values in all isolates were observed to decrease (Figs. 4A and 4B). After 30 h, two different curve patterns were noted: (i) the CI values for all pellicle-forming isolates (AB21, AB34, AB69 and AB97) showed a continuous declination and reached a negative value constant at approximately 35 to 48 h (Fig. 4A) (ii) the CI values for all non-pellicle-forming isolates (AB11, AB20 and AB31) rose until 48 h and reached to CI values of 0.071, 0.068 & 0.077 respectively (Fig. 4B). As shown in Table 2, the statistical analysis showed a significant decrease in CI value (*p* < 0.05) in all four pellicle-forming isolates (AB21, AB34, AB69 and AB97) when compared to their maximum CI value and the CI value at 48 h. However, such a phenomenon was not observed among the non-pellicle-forming isolates (AB11, AB20 and AB31).

**Table 2:** Cell index of pellicle formation among selected clinical carbapenem-resistant *Acinetobacter baumannii* isolates.

Isolates	Cell Index (CI)			*p*-value	Significant difference (*p* < 0.05)
	Highest peak hours		48 h		
AB11	13	0.094 ± 0.015	0.071 ± 0.039	0.525	ns
AB20	12	0.070 ± 0.013	0.068 ± 0.021	0.902	ns
AB31	12	0.088 ± 0.017	0.077 ± 0.032	0.810	ns
AB21	13	0.067 ± 0.010	−0.008 ± 0.014	0.002	^**^
AB34	11	0.034 ± 0.014	−0.051 ± 0.035	0.001	^***^
AB69	11	0.047 ± 0.020	−0.037 ± 0.011	0.001	^***^
AB97	13	0.043 ± 0.001	−0.025 ± 0.040	0.005	^**^

**Notes.**

Footnote: Statistically significant.

** *p* < 0.01.

*** *p* < 0.001.

## Discussion

The capacity of *A. baumannii* to form a pellicle (floating biofilm) at the air-liquid interface is an advantage for their survival in harsh environment settings (Krasauskas et al., 2019). Besides the protection from desiccation in the hospital environment by the extracellular polymeric substances’ matrix of biofilm, the biofilm forms at the air-liquid interface and provides a favourite niche to the aerobic *A. baumannii* because bacteria can obtain nutrients from the liquid media and oxygen directly from air (Nait Chabane et al., 2014). In this study, genotyping results showed that 100% (96/96) of clinical CRAB isolates consist of the genes encoding for the GacSA TCS and this is in agreement that the GacSA TCS is highly conserved in many Gram-negative bacteria (Bhagirath et al., 2019). However, only 4.2% (4/96) isolates (AB21, AB34, AB69 and AB97) demonstrated the ability to form pellicles and these findings agreed with a previous study documented in Australia which showed that pellicle formation is a rare phenotypic trait and was only seen in a limited number of *A. baumannii* (Giles et al., 2015). The authors reported that only 14.8% (8/54) of their tested clinical *A. baumannii* isolates were observed to form robust pellicles. Besides, another study conducted in Southern India also found that only 3.3% (2/60) of multi-drug resistant *A. baumannii* formed thick pellicles (Vijayakumar et al., 2016). Collectively, others’ studies and ours revealed that pellicle formation is not a ubiquitous phenotype for *A. baumannii.* Previous studies reported the involvement of GacA in *A. baumannii* pellicle development and maturation (Marti et al., 2011; Kentache et al., 2017), therefore the four pellicle-forming isolates that harbouring genes encoding for the GacSA TCS in our study warrant for further investigation. Whole genome DNA and RNA sequencing should be carried out to uncover the molecular mechanism involved in CRAB pellicle formation.

Interestingly, all four pellicle-forming isolates in our study were recovered from respiratory-associated samples and none from blood samples, *i.e.,* 1 bronchoalveolar lavage, 1 sputum, 1 tracheal secretion and 1 tracheal swab (Table 3). A similar observation was also reported by Vijayakumar et al. (2016) that two sputum isolates (SP1766 and SP1840) produced thick pellicles but none in blood isolates. It is unclear if pellicle-forming is predominant in the respiratory tract isolates, enhancing their infection or colonisation virulence compared to non-respiratory tract isolates. Therefore, the further characterisation of these four pellicle-forming isolates in the future will provide insights into the pathogenicity of *A. baumannii.*

**Table 3:** Distribution of pellicle-forming isolates among carbapenem-resistant *Acinetobacter baumannii* recovered from respiratory and non-respiratory specimens.

**Sample**	**Type of specimens**	**Isolates**	**Pellicle forming ability (n, %)**	
Respiratory	BAL (Bronchoalveolar lavage)	8	1	12.5
	Respiratory (Tracheal)	5	0	0.0
	Sputum	5	1	20.0
	Tracheal aspirate	6	0	0.0
	Tracheal secretion	38	1	2.6
	Tracheal swab	1	1	100.0
	**Total**	63	4	6.3
Non-respiratory	Blood	16	0	0.0
	Bone	1	0	0.0
	Flank tissue	1	0	0.0
	Gall bladder fluid	1	0	0.0
	Foot tissue	1	0	0.0
	Leg swab	1	0	0.0
	Perianal tissue	1	0	0.0
	Peritoneal fluid	1	0	0.0
	Scrotal tissue	1	0	0.0
	Skin blister swab	1	0	0.0
	Thigh swab	1	0	0.0
	Urine	5	0	0.0
	Wound swab	2	0	0.0
	**Total**	33	0	0.0

Furthermore, our four pellicle-forming isolates and two isolates SP1766 and SP1840 from Vijayakumar’s study exhibited surface-associated motility on semi-solid media, suggesting the involvement of motility in the formation of pellicles. The role of motility in pellicle formation was demonstrated in *Bacillus subtilis* as Δ*hag* and Δ*motAB* showed a lesser collective streak (the accumulation of cell clusters in early maturation of pellicle formation) compared to the wild type, suggesting that the motility enhanced faster colonisation and is essential in pellicle formation (Lee, Rosenberg & Rubinstein, 2019). Although *A. baumannii* lacks flagella and is referred to as a non-motile organism, its surface motility has been described to be mediated by pili (Armitano, Méjean & Jourlin-Castelli, 2014; Marti et al., 2011). Transposon-mediated insertional mutagenesis study demonstrated that the disrupted *bfmR* gene which is responsible for the expression of the *Csu* pili chaperon-usher assembly system abolished the capacity to form floating biofilm (Tomaras et al., 2008). Interestingly, there is a study showed that twitching motility was absent in type IV pili (*pilA*, *pilD*, and *pilT*) mutant strains but not surface-associated motility (Harding et al., 2013). Recently, (Blaschke, Skiebe & Wilharm (2021) reported that 30 novel genes involved in surface-associated motility potentially linked to the pellicle formation of *A. baumannii* and deserved further investigations.

Different types of abiotic surfaces revealed different pellicle-forming capabilities. Our four pellicle-forming isolates cultured in the borosilicate glass tubes (MH: 4/4, LB: 4/4) were observed to have a 50% higher rate of pellicle production compared to polypropylene plastic tubes (MH: 3/4, LB: 1/4), suggesting that these isolates have better bacterial adhesion on glass rather than plastic in this study. This is in accordance with an observation reported by Giles et al. (2015) that 62.5% (5/8) of pellicle-forming strains formed strong pellicles in glass tubes but only 25% (2/8) produced strong pellicles in polypropylene plastic tubes. Moreover, their two clinical *A. baumannii* strains (AYE and AB0057) formed measurable pellicles in glass tubes but failed in polypropylene tubes. On the contrary, several studies showed that pellicle formation of *A. baumannii* was less favourable on glass compared to plastic (McQueary & Actis, 2011; Tomaras et al., 2003). Taken together, these inconsistent results indicate that the capability to form pellicles is not conserved across glass tubes and polypropylene plastic tubes. One of the possible explanations is the heterogeneity amongst *A. baumannii* isolates due to the plastic genome which attributes to the specific adherence strategies such as the production of pili or surface molecules (Antunes, Visca & Towner, 2014; Corral et al., 2021).

Our four pellicle-forming isolates showed a higher rate of pellicle formation in MH medium (Glass: 4/4, Plastic: 3/4) compared to LB medium (Glass: 4/4, Plastic: 1/4), suggesting that nutrient compositions contribute to the pellicle formation. Based on the nutrient compositions, the concentration of nutrients in the MH medium is higher compared to the LB medium. The MH medium consists of beef extract as the source of nitrogen, carbon, and vitamins while the dextrose was used as an energy source. In LB medium, the peptone provides a nitrogen source while yeast extract plays a role in basic nutrients, growth factors, vitamins, amino acids and trace elements (Bonnet et al., 2019). A biofilm formation study of *Pseudomonas* spp. also reported to have a higher biofilm formation rate in MH broth (99/100) over LB medium (92/100) and Tryptic Soy Broth medium (96/100) at 24 h (Robinson et al., 2019). Furthermore, the casamino acids (a complex mixture of amino acids and small peptides) which are similar to the casein hydrolysates in the MH medium were reported to play a crucial role in the regulation of expression in the curli in *Salmonella* that consequently promotes pellicle formation (Paytubi et al., 2017). Recently, two studies reported that the biofilm formation on solid surfaces by environmental *A. baumannii* is more favourable in nutrient-poor media (Eze & El Zowalaty, 2019; Kizny Gordon et al., 2017). Taken all together, different nutrient compositions in different media might affect the expression of genes in promoting different types of pellicle/biofilm formation in *A. baumannii*.

The introduction of impedance-based xCELLigence RTCA technology allowed us to further characterise the pellicle formation in real-time and understand the behaviour of pellicle-forming CRAB isolates that were missed out from the endpoint result of the crystal violet assay. The CI curve obtained in our study can be distinguished into four stages that correlate to the pellicle formation developmental phases including attachment, conversion, growth and maturation (Lee, Rosenberg & Rubinstein, 2019). At 0 to 4 h (Stage I), the negative CI values were observed after bacterial seeding onto the E-plate, suggesting the bacterial cell is entering an irreversible attachment stage. At 4 to 13 h (Stage II), an increase of CI values showing response to the detected robust impedance signal signifies the increase of bacterial cell density. The trend of CI values for pellicle and non-pellicle-forming isolates starts to differ from stage III to stage IV.

For pellicle-forming isolates, the steep decrease rate of CI values after reaching their maximum values at 13 h until 30 h (stage III) was observed, indicating that these bacteria entered the growth stage of pellicle development. The streak bacteria migrate towards the liquid-air interface, thus the bacterial density in the broth is expected to be reduced, hence corresponding to the decrease of impedance. A similar observation was also reported by van Duuren et al. (2017) in their *P. aeruginosa* pellicle study using impedance-based spectroscopy. The authors suggested that the formation of pellicles may increase the capacitance of the liquid medium and be attributed to the decrease in impedance. Therefore, the growth phase of pellicle formation in our four CRAB isolates is suggested to start after 13 h. From 30 h onwards (stage IV), the declination of CI values was observed to a halt and this suggested that the growth phase of pellicle formation has reached its maturation phase hence there was low bacterial density in the liquid medium. For non-pellicle-forming isolates, the slow decrease rate of CI values after their maximum CI at 13 h until 30 h (stage III) suggested the detachment of the sedimented bacterial cells from the surface of microelectrodes into the medium suspension has occurred and followed by a slow dispersion (stage IV). Overall, these observations suggest that the RTCA method is sensitive in detecting the differences among pellicle and non-pellicle-forming isolates. Further mapping of the regulatory process during different stages of pellicle formation tracked in real-time remains to be elucidated. In addition, other factors including cell surface hydrophobicity, oxygen sensing or aerotaxis and involvement of surfactant should be examined to provide a more comprehensive understanding of pellicle development in these identified four CRAB pellicle-forming isolates.

## Conclusions

All 96 clinical CRAB isolates carried the *gacS* and *gacA* genes while only four isolates displayed the ability of pellicle-formation phenotypically. These four pellicle-forming isolates formed more robust pellicles in borosilicate glass tubes using the nutrient-richer MH medium. The impedance-based RTCA showed distinctive results between pellicle-forming and non-pellicle-forming CRAB isolates. As these four CRAB isolates could be potentially more virulent, further study is warranted to provide insights into their pathogenic molecular mechanisms.

##  Supplemental Information

10.7717/peerj.15304/supp-1 Supplemental Information 1 Bacterial migration diameter from inoculation centre on 0.5% (w/v) Bacto™agar The values of diameter shown are mean ± SD of triplicate experiments. There were statistically significant differences in the migration diameter of each pellicle forming isolates (AB21, AB34, AB69 & AB97) and compared to the average of non-pellicle forming isolates (AB11, AB20 & AB31) (** *p* < 0.01, **** *p* < 0.0001) with the Dunnett’s multiple comparison test of one-way analysis of variance (ANOVA) test.

10.7717/peerj.15304/supp-2 Supplemental Information 2 Raw data

## Additional Information and Declarations

### Data Availability

The following information was supplied regarding data availability: The raw data are available in the Supplemental File.
