# Supplementary material for: Characterisation of pellicle-forming ability in clinical carbapenem-resistant Acinetobacter baumannii
Source: PeerJ. 2023 May 15;11:e15304. doi: 10.7717/peerj.15304 (PMC10194081; doi:10.7717/peerj.15304)
Supplement: Supplemental Information 1 — The values of diameter shown are mean ± SD of triplicate experiments. There were statistically significant differences in the migration diameter of each pellicle forming isolates (AB21, AB34, AB69 & AB97) and compared to the average of non-pellicle forming isolates (AB11, AB20 & AB31) (** p < 0.01, **** p < 0.0001) with the Dunnett’s multiple comparison test of one-way analysis of variance (ANOVA) test. [file peerj-11-15304-s001.docx]

**Table S1: Bacterial migration diameter from inoculation centre on 0.5% (w/v) Bacto™ agar.** The values of diameter shown are mean ± SD of triplicate experiments. There were statistically significant differences in the migration diameter of each pellicle forming isolates (AB21, AB34, AB69 & AB97) and compared to the average of non-pellicle forming isolates (AB11, AB20 & AB31) (***p* <0.01, **** *p* <0.0001) with the Dunnett’s multiple comparison test of one-way analysis of variance (ANOVA) test.

|  | **Isolates** | **Diameter (mm)** | ***p-value*** |
| --- | --- | --- | --- |
| **Pellicle forming strains** | **AB21** | 55.2 ± 7.9 | <0.0001 |
|  | **AB34** | 50.2 ± 5.3 | <0.0001 |
|  | **AB69** | 48.0 ± 4.9 | <0.0001 |
|  | **AB97** | 18.2 ± 1.7 | 0.0021 |
| **Non-pellicle forming strains** | **AB11** | 12.1 ± 0.6 | - |
|  | **AB20** | 13.5 ± 1.2 | - |
|  | **AB31** | 11.6 ± 0.5 | - |
|  | **Average** | 12.4 ± 1.2 | - |
| **Positive control** | ***Salmonella typhimurium* ATCC 14028** | 85.0 ± 0.0 | - |
| **Negative control** | ***Klebsiella pneumoniae* ATCC 700603** | 11.3 ± 0.5 | - |
